# Risk Factors of Mortality from All Asbestos-Related Diseases: A Competing Risk Analysis

**DOI:** 10.1155/2017/9015914

**Published:** 2017-06-07

**Authors:** Rafael Abós-Herràndiz, Teresa Rodriguez-Blanco, Isabel Garcia-Allas, Isabel-Magdalena Rosell-Murphy, Constança Albertí-Casas, Josep Tarrés, Illona Krier-Günther, Xavier Martinez-Artés, Ramon Orriols, Isidre Grimau-Malet, Jaume Canela-Soler

**Affiliations:** ^1^Catalan Health Institute (ICS), Division of Primary Health Care, Department of Health, Barcelona, Catalonia, Spain; ^2^Primary Care Research Institute (IDIAP Jordi Gol) and Research Associate, Autonomous University of Barcelona (UAB), Cerdanyola del Vallès, Spain; ^3^Catalan Institute for Medical Evaluation (ICAM), Barcelona, Catalonia, Spain; ^4^Pneumology Unit, Hospitals de Girona i Salt, Institut d'Investigació Biomèdica de Girona (IDIBGI), Girona, Catalonia, Spain; ^5^Ciber de Enfermedades Respiratorias (CIBERES), Palma de Mallorca, Baleares, Spain; ^6^Palliative Care Unit, Parc Taulí Hospital, Barcelona, Spain; ^7^Department of Public Health, University of Barcelona (UB), Barcelona, Spain; ^8^Department of Epidemiology and Biostatistics, College of Public Health, University of South Florida, Tampa, FL, USA

## Abstract

**Background:**

The mortality from all malignant and nonmalignant asbestos-related diseases remains unknown. The authors assessed the incidence and risk factors for all asbestos-related deaths.

**Methods:**

The sample included 544 patients from an asbestos-exposed community in the area of Barcelona (Spain), between Jan 1, 1970, and Dec 31, 2006. Competing risk regression through a subdistribution hazard analysis was used to estimate risk factors for the outcomes.

**Results:**

Asbestos-related deaths were observed in 167 (30.7%) patients and 57.5% of these deaths were caused by some type of mesothelioma. The incidence rate after diagnosis was 3,600 per 100,000 person-years. In 7.5% of patients death was non-asbestos-related, while pleural and peritoneal mesothelioma were identified in 87 (16.0%) and 18 (3.3%) patients, respectively.

**Conclusions:**

Age, sex, household exposure, cumulative nonmalignant asbestos-related disease, and single malignant pathology were identified as risk factors for asbestos-related death. These findings suggest the need to develop a preventive approach to the community and to improve the clinical follow-up process of these patients.

## 1. Introduction

Asbestos is an established human carcinogen [1] found naturally in rocks and widely used by industry [2]. The exposure to this material occurs through the inhalation of fibres in the ambient air in factories handling asbestos or in the indoor air in housing and buildings containing asbestos materials [3]. First published at the beginning of the 20th century, its harmful effects received the ARD acronym (asbestos-related disease) and, at the beginning of the 21st century, ARD became a relevant public health issue [4].

According to various estimates [5, 6], Spanish imports of this material during the 20th century were 2.6 million MT, with a peak in 1974 (126.000 MT), corresponding to a lower consumption in more developed economies [6, 7]. In Spain there is a lack of adequate estimates of the number of workers exposed to asbestos and community-based registries of ARD-affected people [6]. In this context, an important public health issue concerns the risk of developing ARD from environmental asbestos exposure in residents who live near big asbestos processing plants [8–10].

All types of asbestos can cause mesothelioma, lung cancer [1], and nonmalignant ARD such as asbestosis and pleural thickness [3, 4, 9]. There is a concern about the potential mortality of ARD due to malign mesothelioma [7, 11] and although infrequent, it is one of the most lethal diseases among this group of pathologies.

Consistent evidence of the harmful effects of asbestos [2, 12, 13] and trade restrictions introduced in Europe in the 1980s onwards have contributed to its declining use in some countries over the past decades. However, the asbestos latent period most commonly reported of 20–40+ years from the beginning of exposure [11, 14] means that in case of malign pleural mesothelioma, incidence rates rose steeply until the 1990s in most European countries and the United States. This continued increase over the past two decades in industrialised countries is yet to peak worldwide [2, 3, 7, 15, 16].

About 125 million workers continue to be directly exposed to asbestos in the world [3, 17]. In 2004, asbestos-related lung cancer, malignant pleural mesothelioma, and asbestosis from occupational exposure have resulted globally in more than 100,000 deaths. Although several thousands of deaths can be attributed to other ARD or to nonoccupational exposure to asbestos [3], asbestos mortality has not been considered taking into account non-ARD death as a competing risk. Furthermore, the overall impact on the whole population, in terms of mortality for all forms of ARD and particularly the benign pathology, remains understudied [16]. Therefore, the main objective of this study is to assess incidence and independent risk factors of mortality from all forms of ARD with a competing risk analysis in a cohort of primary care patients. The patients were identified from 1970 to 2006 in the region of Barcelona (Spain).

## 2. Materials and Methods

### 2.1. Design, Setting, and Study Population

This is a community-based cohort study with catchment population of 485,578 people (2007 Census) assigned to 14 Primary Care Centers in a low socioeconomic area (according to the Gross Domestic Product per capita, 2012) [18] of the region of Barcelona (Spain). A cement processing plant remained in this zone for 90 years (1907–1997). This facility processed 14.446 MT of asbestos per year (80% white, 15% blue, and 5% brown) which corresponds to a third of the entire volume of asbestos imported into Spain during the 20th century [10].

Since 2003, a primary healthcare research team has launched a public-funded research program in coordination with the reference hospital to analyse the profile of ARD events [19]. The detailed research protocol has been already published [20]. The study cohort included all patients, 544, with any ARD diagnosis identified between Jan 1st, 1970, and Dec 31st, 2006, in the catchment area.

### 2.2. Asbestos Health Effects Diagnosis Criteria and Study Case Definition

The ARD acronym group comprises 10 heterogeneous entities [21, 22] to describe asbestos health effects. The malignant pathology includes pleural mesothelioma, peritoneal mesothelioma, bronchopulmonary neoplasm, and several rare lung neoplasm. The nonmalign (benign) pathology comprises pleural plaques, pleural thickness, pulmonary effusions or hydrothorax, rounded atelectasis, lung fibrosis (asbestosis), and chronic airway obstruction.

We defined ARD diagnosis criteria [21] as having the next three clinical conditions: (a) past history of asbestos exposure and/or a reasonable ARD latent time, (b) evidence of asbestos exposure by clear structural damage in the lung confirmed by rx-chest and/or evidence of asbestos bodies (identified by bronchoalveolar lavage or cytology), and (c) exclusion of other more prevalent diseases, such as tuberculosis. The pathological anatomy diagnosis prevailed over clinical suspicion. All diagnoses were confirmed by a general practitioner and a specialist in respiratory medicine. All ARD deaths were registered using the tenth International Classification of Diseases (10th ICD) but 8th ICD and 9th ICD versions were also required (Table 1).

### 2.3. Data Sources

Data were retrospectively collected from Jan 1, 1970, until Dec 31, 2003, and prospectively collected from this date. Before 1970, most community-based data and clinical information available were unreliable.

Clinical information was provided by primary healthcare and tertiary hospital medical records (morbidity, mortality, and discharge). Face-to-face interviews with patients or their relatives were also carried out to complete the information and to identify the source and duration of asbestos exposure. In case of any combined sources of exposure, the patient was assigned to the highest exposure group according to the following order: labor, household, and environmental. In case of work exposure, work contracts and/or payroll were required; in case of household exposure, a history of living with a worker exposed to asbestos in his/her job was required; and for environmental exposure, the main resident had to be located within the study area. The length of occupational exposure to asbestos was confirmed with work contracts and/or available payroll. The onset of exposure for each patient was identified when the patient started working in the factory, coexisting with a worker or living in the study area.

### 2.4. Outcome Variable

The primary outcome was considered ARD-related death, death due to ARD, confirmed with the official mortality registry data bank in all cases. Moreover, all our deaths were linked with this register, and the concordance rate between our underlying cause of death and the mortality registry was 97.5%.

### 2.5. Covariates

The following categorical covariates were considered: sex (female/male), age (<50, 50–60, 61–70, 71–80, >80 years), smoking status (nonsmoker, smoker, ex-smoker, and missing), number of ARD diagnosed per person (1 benign, 2 or 3 benign, 4 or 5 benign/at least 1 malignant), and source of exposure (environmental, household, and labor). The number of ARD categories is based on a quantitative approach according to a biological gradient presented as a scale [23].

We categorised length of exposure according to the quartiles of the study population [0–11.3), [11.3–20.9), [20.9–30.6), (>=30.6 years). The smoking status was not fully collected and a missing category was created for the statistical analysis. All the above covariates were measured at the date of diagnosis. We also recorded date of death, date(s) of any ARD events diagnosed, and their clinical-related signs and symptoms.

### 2.6. Statistical Analysis

Descriptive statistics were used to summarize the overall information. Categorical variables were expressed as numbers (percentages) and continuous variables as means (standard deviation, SD) and as medians (interquartile ranges, IQR). Incidence rates (IR) of death were calculated as person-years. For the numerator we used number of deaths. The denominator was the sum of the person-time contributed for each individual. Kaplan–Meier life tables were used to calculate the quartiles of survival rates across the strata of gender and age groups [24]. Log-rank and Breslow tests were used for testing the equality of survival curves among strata of these covariates. Competing risk regression through a subdistribution hazard analysis [25] was applied to estimate the association between risk factors and the time to ARD-related death. The subdistribution hazard ratio (SHR) reflects both the association of a covariate with the event of interest and the contribution of the competing events by actively maintaining individuals in the risk sets (instead of being censored). Effects on the SHR can be directly translated to effects on the cumulative incidence function. The SHR is the relative change in the hazard for one category of a covariate compared with the reference category of the corresponding covariate. A SHR > 1 indicates that those with a higher value of a covariate will have a quicker time to event in the study population. Similarly, a SHR < 1 indicates a longer time to event for those with a higher value of a covariate [26]. The SHR was estimated as the exponential function of the regression coefficient, that is, exp (coefficient) through the Stata command stcrreg. The follow-up time, in years, was the time scale. The beginning of the survival time, when a subject becomes at risk, was the onset of exposure. Each subject's follow-up time began after the date of diagnosis and ended the date of any ARD death or censoring. Therefore, the analysis was corrected for delayed entry, such that individuals were considered under observation of ARD death only from the date of diagnosis. Their entry into the risk sets was delayed. There was a selection process taking place in that only those subjects who were diagnosed were eligible to be included in the study [27]. The latent period was defined as the time between the date of the onset of exposure to asbestos and the date of the diagnosis.

A competing risk event was considered when any other causes of death (non-ARD) occurred without a preceding outcome. The source and length of exposure have been judged epidemiologically relevant variables, being included in the final model. Additional analysis (not shown) was performed, imputing tobacco missing values by multiple imputation methods. The results were of a similar magnitude. We checked for interactions but no significant ones were found. The final model was adjusted by all significant, clinical, and confounder variables. The proportional hazard assumptions were assessed by adding the covariate by log-time interactions to the model [25]. All results were expressed with 95% confidence intervals (CIs) and statistical significance was set at *P* < 0.05 (two-tailed). The analyses were performed using Stata/SE Version 12.1 (Stata Corp. LP, College Station, TX, USA).

### 2.7. Ethical Conditions

We conducted our research according to the tenets established by the Declaration of Helsinki and Spanish Good Research Practice Guidelines. The study protocol was approved by the Clinical Research Ethics Committee of the Institute-IDIAP Jordi Gol. Participants were given written information and were informed about implications of the study prior to consenting to participate. Confidentiality and anonymity were according to Spanish Personal Data Protection Laws [28].

## 3. Results 

Between the 544 cohort members, 73.2% of the subjects were male, with a mean age of 63.7 years (SD: 12.2). The 41.1% of the total cohort suffered more than one benign ARD and 25.7% had at least one malignant ARD. The most prevalent source of exposure was from labor (73.5%) followed by environmental exposure (15.3%). The median length of exposure was 21.0 years (IQR: 11.0–30.5) and the median length of latency was 41.6 years (IQR: 30.0–52.2) (Table 2). Among workers, 77.1% had at least one malignancy and, in the case of environmental and household exposure, the proportion was 11.4%.

Pleural mesothelioma was diagnosed in 84 (15.4%) patients; peritoneal mesothelioma in 15 (2.8%); and both pleural and peritoneal mesotheliomas were found in 3 (0.6%) patients. The remaining 38 malignancies were bronchopulmonary neoplasms. All cases of peritoneal mesothelioma were men under 80 years, whose source of exposure was their employment. The median survival time after the diagnosis of pleural mesothelioma was 7.4 months and 8.4 months in peritoneal.

Benign diseases diagnosed were pleural plaques (436), pleural thickening (218), pulmonary effusions (58), rounded atelectasis (25), and lung fibrosis (221). During the follow-up (median: 4.1 years), ARD-related death was observed in 167 (30.7%) patients, 142 (26.1%) in men. Of these deaths, 78 (46.7%) were due to pleural mesothelioma, 15 (9%) to peritoneal mesothelioma, 3 (1.8%) to both, 31 (18.5%) to lung cancers, 1 (0.6%) to bronchopulmonary cancer, and 39 (23.4%) to at least one benign ARD (29 fibrosis, 10 pleural plaques, 6 pleural thickening, 3 atelectasis, and 5 benign pleural effusions).

Non-ARD deaths were 41 (7.5%), of which 36 (87.8%) were men and five (12.2%) were women. The incidence rate of ARD-related death after the diagnosis was 3600 per 100,000 person-years and the incidence rate of ARD-related death after the start of exposure was 620 per 100,000 person-years (men: 760, women: 304). The median time of survival from the start of exposure was 40.5 years; 40.9 years for males and 35.5 years for females. Moreover, the difference in survival curves from ARD between age groups was highly significant (Log-rank/Breslow test: *P* < 0.001). The incidence rate of ARD mortality after the diagnosis increased with age (Table 3).

In the adjusted analysis of ARD death, the model showed evidence of nonproportional hazard in age and gender. Men presented an increased and significantly higher risk than women of dying from ARD, after 40 years from the start of asbestos exposure. Patients aged between 60 and 69 years had a higher risk of death from ARD in the first 30 years of exposure, followed by the group 70–79 years (Table 4).

Former smokers were associated with a lower risk of death from ARD-related diseases as compared with nonsmokers and people with household exposure were at higher risk than those with labor, if we compared to those with environmental exposure.

The ARD mortality risk rose sharply with the increasing number of benign ARD, presenting the highest risk in patients with at least one malignant ARD (HR = 128.3; 95% IC: 3 (61.8–266.33)) (Table 5).

## 4. Discussion

### 4.1. Summarizing Key Results with Reference to Study Objectives

Our study is the first to analyse the risk of death for all ARD in a community. The main result is that nearly a third of patients diagnosed with ARD die from it and more than half due to some form of mesothelioma. Our research shows that the following have a higher risk of dying from ARD: (a) person between 60 and 80 years of age during the first 30 years of exposure to asbestos, (b) men with more than 40 years of exposure to this material, (c) people cohabiting with an asbestos worker, and (d) an individual being diagnosed with four or more ARD benign entities or at least one that is malignant.

### 4.2. Comparisons

The high overall incidence of all ARD-related deaths in a community, which increases with age, is hardly comparable with current literature. This could be because our study has estimated that rate from the time of clinical diagnosis of ARD and not from the start of asbestos exposure. On the other hand, the analysis considered all deaths caused by ARD and not by any specific ARD.

Our study shows that men have a higher risk of death from an ARD after 40 years of asbestos exposure, in agreement with other published studies [29]. It is likely that females with occupational exposure could have worked in the factory offices, so that their levels of asbestos exposure were lower than men. Besides, older patients could be more likely to die due to more comorbidity. In contrast, and similarly to other studies in women exposed to asbestos from environmental [30] or household exposure [31, 32], our research shows that women have a higher risk of death during the first 40 years of exposure compared to men. However, this risk is not statistically significant.

In our research, the proportion of deaths is much higher in men than in women during the same period (male: 44.7% versus female: 20.5%) as shown by Helland et al. [33]. It is not possible to rule out underreporting in all ARD entities, not only in the first 40 years, but also throughout the study period, which would produce a certain underestimation of the risk of death from ARD, especially in men.

The greatest risk of dying from ARD in the household group similarly observed by Ferrante et al. [32] can be explained by the healthy survival effect. This is due to the long duration of exposure under conditions of lower susceptibility to ARD in patients who have survived. This risk could be higher since forms of ARD other than mesothelioma are difficult to detect in nonoccupational exposure.

It is difficult to compare our data on the benign fraction of ARD in Spain since the publication of the series of these entities in the occupational field of asbestos should have been more thorough. Notably between 1990 and 2010 only 535 cases of a single labor benign ARD affecting 98% of men were declared [6].

Benign ARD is a common occurring abnormality in people exposed to asbestos and is not a precursor of malignant ARD [29, 34] and our study coincides with this [19]. In spite of this benignity [29, 34] our cutting-edge research shows that there is an increased risk of dying from an accumulated number of benign entities (HR > 10 for 4 or 5 benign entities) or having at least one that is malignant (HR = 128.3).

Regarding malignant ARD, it is known that the median survival time is a good point estimate of mortality from ARD [33, 35, 36]. Our data on this median is similar to those reported by other authors; Lee and colleagues put it at 6 months [35] other researchers at 9.3 months in Norway [33] and 18 months in Turkey [36].

### 4.3. Strengths of the Study

The cohort in the present study is unique because it includes an overall and a well-defined population in an area with high and long asbestos exposure with a similar latent period to the asbestos literature [32]. The completeness of the data was checked centrally and the outcomes were ascertained and confirmed after a review of medical records. Moreover, the concordance rate between our underlying causes of death and those from the official mortality register data bank was high.

We adjusted HR for competing risks of death to avoid overestimation of the risk of ARD events, considering that the time at risk is long and competing risks of death from other causes are high. Moreover, the subdistribution of hazard is an appropriate measure for predicting an individual's risk for an outcome or allocating resources. This method models the effect of covariates on the event by incorporating the association between covariates and the event of interest as well as the competing event which influences the risk set [26].

### 4.4. Limitations of the Study

In the absence of official measures of occupational and environmental asbestos and general data on the overall ARD morbidity and mortality in Spain [6], the duration of exposure has been employed as a surrogate quantitative exposure measure. We have not considered neither the intensity of exposure that could have varied across individuals [30] and time periods nor the recall bias of past exposures that could have caused biased estimates of association [37]. We have not quantified the effect of asbestos waste sites in an area free of naturally occurring asbestos [38, 39], which holds the highest Spanish mortality rate from mesothelioma [40]. The information related to any other ARD deaths (benign or malign) remains unknown. We have not taken into account variables such as physical examination interval or clinical treatment due to irregular follow-up and lack of medical record information.

The diagnosis of ARD can lead to misclassification errors because pleural plaques can be valued as mimicking shadows [41], due to a lack of sensitivity in chest radiographs [42]; pulmonary fibrosis shows a clear separation between its clinicoradiology and histopathology [9] and the three histological types of pleural mesothelioma may have different clinical outcomes [43]. Underestimation of exposure must be considered due to the clearance of asbestos in the body and the high cost of pathological anatomy techniques for the detection of asbestos bodies. It should be noted in the interpretation of results that the definition of ARD includes a heterogeneous group of malignant and benign diseases. Moreover, the existing confidentiality in the Spanish labor regulation for people working with asbestos, the usual refusal to participate in such studies, the healthy survivor effect, and a misclassification error in the cause of death [16, 32] could have all caused a selection bias. These biases may underestimate the risk of death from all ARD. Other etiologic factors such as the genetic profile and the different susceptibility to asbestos exposure have not been studied in this research.

The aggregate analysis of ARD deaths using competing risks allows a better approximation to the population estimate of the risk from exposure to asbestos. In spite of this, its result can only be generalizable for similar competing risk rate communities [26].

### 4.5. Overall Interpretation and Implications for Research, Practice, and Prevention

Our research shows the effects of prolonged exposure to asbestos which resulted in a high incidence rate of ARD mortality. This kind of exposure and outcome can only be produced by inadequate preventive and protective measures against asbestos and a permissive legislation used and marketed until 2001. The underreporting of benign asbestos occupational morbidity and the accumulation of benign ARD mortality could have contributed to these results. It is necessary to develop a preventive approach to the community [44], create a registry of people exposed to asbestos and ARD cases, and improve the clinical follow-up of all ARD patients.

Globally, the annual market demand for asbestos exceeds 2,000,000 MT [4, 45] and several authors have predicted a revival of ARD in the coming decades, especially in low-income countries [2, 9] despite the worldwide ARD pandemic [4]. Undoubtedly, the absence of an international government health platform dedicated to the study and control of the effects of asbestos removes the possibility of eradicating death by ARD [46, 47].

### 4.6. In Summary

The harmful effects of a community permanently exposed to asbestos reflect that age, sex, nonoccupational exposure, cumulated benign ARD, and single malign pathology account as risk factors for a high risk of death from ARD. A clinical and preventive approach should be implemented in the community in order to control the effects of the asbestos.

## Figures and Tables

**Table 1:** International death codes for asbestos-related diseases (ARD).

ARD event	IDC-8	IDC-9	IDC-10
Pleural mesothelioma	—	—	C450
Peritoneal mesothelioma	—	—	C451
Unspecified mesothelioma	—	—	C459
Malign pleural Neoplasm	1630	163	C384
Asbestosis	5152	501	J61
Pleural plaques	—	—	J920
Unspecified pleural disease	5110	5110	J948
Unspecified fibrothorax	5110	5110	J941
Hydrothorax	5112	5118	J90
Rounded atelectasis unspecified	5190	5180	J981
Pneumoconiosis	5159	505	J64
Chronic restricted aerial flow	—	—	—

*Note*. IDC: International Disease Code.

**Table 2:** Demographic and clinical characteristics at the date of diagnosis of ARD, Barcelona (Spain), 1970–2006 (*N* = 544).

Characteristics	Number (%)
Gender, male	398 (73.2)
Age (yrs); mean (SD)	63.7 (12.2)
Age groups	
<50	82 (15.1)
50–59	139 (25.6)
60–69	134 (24.6)
70–79	140 (25.7)
>=80	48 (9.0)
Number of ARD	
1 benign	180 (33.1)
2 or 3 benign	201 (36.9)
4 or 5 benign	23 (4.2)
>=1 malign	140 (25.7)
Source of exposure	
Environmental	83 (15.3)
Household	61 (11.2)
Labor	400 (73.5)
Length of exposure (yrs); mean (SD); median (IQR)	22.9 (15.8); 21.0 (11.0–30.5)
[0–11.3)	130 (23.9)
[11.3–20.9)	142 (26.1)
[20.9–30.6)	136 (25.0)
>=30.6	136 (25.0)
Period of latency (yrs); mean (SD); median (IQR)	41.0 (15.6); 41.6 (30.0–52.2)
Smoking status	
Nonsmoker	207 (38.1)
Smoker	153 (28.1)
Former	137 (25.2)
Missing	47 (8.6)
Age at onset of exposure of patient with outcomes (yrs); mean (SD)	22.7 (11.7)
ARD death (*n* = 167)	26.0 (10.4)
Pleural mesothelioma diagnosis (*n* = 87)	23.2 (11.9)
Peritoneal mesothelioma diagnosis (*n* = 18)	27.4 (5.7)

ARD: asbestos-related disease; SD: standard deviation; IQR: interquartile range.

**Table 3:** Characteristics of ARD death overall and by gender and age group, Barcelona (Spain), 1970–2006 (*N* = 544).

Outcomes	Number of events (%)	Follow-up time (yrs)^*∗*^; mean (SD); median (IQR)	Person-years at risk	Incidence rate/100000 person-years (95% CI)	Log-rank/Breslow test; *P* values
Death from all ARD					
Overall	167 (30.7)	8.5 (9.1); 4.1 (1.5–13.7)	4611.4	3600 (3092–4191)	
Gender					
Men	142 (26.1)	9.4 (9.8); 4.8 (1.6–16.2)	3746.2	3764 (3191–4439)	0.073/0.164
Women	25 (4.6%)	5.9 (6.4); 2.8 (1.3–9.0)	865.2	2890 (1952–4276)	
Age groups					
<50	30 (5.5%)	18.9 (10.4); 21.5 (12.2–28.5)	1551.4	1934 (1352–2766)	<0.001/<0.001
50–59	43 (7.9%)	11.9 (9.6); 10.0 (2.9–19.5)	1652.6	2602 (1930–3508)	
60–69	40 (7.4%)	6.3 (6.4); 3.6 (1.4–9.8)	846.4	4726 (3466–6443)	
70–79	42 (7.7%)	3.2 (3.2); 2.4 (0.9–4.6)	452.3	9065 (6674–12311)	
>=80	12 (2.2%)	2.2 (1.9); 1.8 (0.8–2.8)	108.6	11045 (6273–19449)	

ARD: asbestos-related diseases; SD: standard deviation; IQR: interquartile range; CI: confidence interval. ^*∗*^The follow-up time for death from all ARD was defined as the number of years from the date of diagnosis to the date of any ARD-related-death or to censoring.

**Table 4:** Adjusted effects of nonproportional risk factors on subhazard from ARD death, Barcelona (Spain), 1970–2006 (*N* = 544).

Survival time (yrs)	Sex SHR (95% CI)^&^ (Ref: female)	*P* value	Age groups (Ref: <50 yrs)	SHR (95% IC)^&^	*P*value
15	0.34 (0.09–1.29)	0.114	50–59	2.01 (0.42–9.66)	0.381
			60–69	37,17 (6.69–206.58)	0.000
			70–79	11.02 (2.00–60.62)	0.006
			>=80	5.85 (0.41–83.47)	0.193
20	0.43 (0.13–1.36)	0.149	50–59	1.61 (0.43–6.00)	0.477
			60–69	20.10 (4.84–83.43)	<0.001
			70–79	8.26 (1.95–35.04)	0.004
			>=80	5.07 (0.51–50.78)	0.167
30	0.67 (0.30–1.53)	0.342	50–59	1.03 (0.43–2.49)	0.947
			60–69	5.88 (2.39–14.47)	<0.001
			70–79	4.64 (1.80–12.00)	0.002
			>=80	3.82 (0.75–19.39)	0.106
40	1.05 (0.60–1.84)	0.852	50–59	0.66 (0.32–1.35)	0.253
			60–69	1.72 (0.90–3.29)	0.102
			70–79	2.61 (1.48–4.60)	0.001
			>=80	2.87 (1.00–8.25)	0.050
50	1.66 (1.02–2.69)	0.042	50–59	0.42 (0.16–1.10)	0.078
			60–69	0.50 (0.20–1.27)	0.144
			70–79	1.47 (0.82–2.64)	0.202
			>=80	2.16 (0.92–5.07)	0.077
60	2.60 (1.32–5.11)	0.006	50–59	—	—
			60–69	0.15 (0.03–0.63)	0.010
			70–79	0.82 (0.31–2.21)	0.700
			>=80	1.62 (0.48–5.48)	0.434
70	4.08 (1.53–10.92)	0.005	50–59	—	—
			60–69	—	—
			70–79	0.46 (0.10–2.04)	0.309
			>=80	1.22 (0.19–7.66)	0.830
80	6.41 (1.69–24.26)	0.006	50–59	—	—
			60–69	—	—
			70–79	—	—
			>=80	0.69 (0.07–11.54)	0.948

*Note*. Ref: reference; CI: confidence interval; SHR: subdistribution hazard ratio. ^&^Cox proportional hazard model, accounting for other causes of death as competing risks. Robust standard errors. All SHRs were adjusted for sex, age groups, smoking status, source of exposure, length of exposure, number of ARD, and sex*∗*time and age groups*∗*time interactions.

**Table 5:** Adjusted effects of proportional risk factors on subhazard from ARD death, Barcelona (Spain), 1970–2006 (*N* = 544).

Predictors	SHR (95% CI)^&^	*P* value
Smoking status		
Nonsmoker (Ref.)	1	0.003
Smoker	1.01 (0.59–1.71)	
Former	0.56 (0.35–0.87)	
Missing	1.79 (0.98–3.26)	
Source of exposure		
Environmental (Ref.)	1	0.074
Household	1.98 (1.07–3.64)	
Labor	1.22 (0.68–2.19)	
Length of exposure (yrs)		
[0–11.3) (Ref.)	1	0.935
[11.3–20.9)	1.17 (0.70–1.97)	
[20.9–30.6)	1.20 (0.63–2.26)	
>=30.6	1.18 (0.58–2.42)	
Number of ARD		
1 benign (Ref.)	1	<0.001
2 or 3 benign	1.94 (0.86–4.38)	
4 or 5 benign	10.28 (3.36–31.43)	
At least 1 malign	128.30 (61.82–266.25)	

*Note*. Ref: reference; SHR: subdistribution hazard ratio; CI: confidence interval; ^&^Cox proportional hazard model, accounting for other causes of death as competing risks. Robust standard errors. All SHRs were adjusted for the other covariates in the table and sex, age groups, and sex*∗*time and age groups*∗*time interactions

## Abbreviations

ARD: Asbestos-related diseases
ICD: International code classification
MT: Metric tonnes
SD: Standard deviation
IQR: Interquartile ranges
IR: Incidence rate in person-years per 100,000
Kms: Kilometers
SHR: Subdistribution hazard ratio
95% CIs: 95% confidence Intervals
*P*: *P* value
IDIAP: Institut D'Investigació en Atenció Primaria.

## References

[1] International Agency for Research on Cancer (1987). Overall evaluations of carcinogenicity: an updating of IARC monographs. *IARC Monographs on the Evaluation of Carcinogenic Risks to Humans*.

[2] Peto J., Decarli A., la Vecchia C., Levi F., Negri E. (1999). The European mesothelioma epidemic. *British Journal of Cancer*.

[3] (July 2010). Preventing diseases through healthy environments. http://apps.who.int/iris/bitstream/10665/204585/1/9789241565196_eng.pdf?ua=1.

[4] Stayner L., Welch L. S., Lemen R. (2013). The worldwide pandemic of asbestos-related diseases. *Annual Review of Public Health*.

[5] Artieda L., Beloqui A., Lezaun M. (2005). Cohorte poblacional de trabajadores expuestos a amianto: Navarra 1999-2004. *Anales del Sistema Sanitario de Navarra*.

[6] Garcia M., Menéndez A., Castañeda R. (2012). Incidencia en España de la Asbestosis y otras enfermedades pulmonares benignas debidas al amianto durante el período 1962-2010. *Rev Esp Salud Pública*.

[7] Peto J., Matthews F. E., Hodgson J. T., Jones J. R. (1995). Continuing increase in mesothelioma mortality in Britain. *The Lancet*.

[8] Magnani C., Mollo F., Paoletti L. (1998). Asbestos lung burden and asbestosis after occupational and environmental exposure in an asbestos cement manufacturing area: a necropsy study. *Occupational and Environmental Medicine*.

[9] Kamp D. W. (2009). Asbestos-induced lung diseases: an update. *Translational Research*.

[10] Tarrés J., Albertí C., Martínez-Artés X. (2013). Pleural mesothelioma in relation to meteorological conditions and residential distance from na industrial source of asbestos. *Occupational and Environmental Medicine*.

[11] Ray M., Kindler H. L. (2009). Malignant pleural mesothelioma: an update on biomarkers and treatment. *Chest*.

[12] Selikoff I. J., Churg J., Hammond E. C. (1964). Asbestos exposure and neoplasia. *JAMA: The Journal of the American Medical Association*.

[13] Selikoff I. J., Churg J., Hammond E. C. (1964). Asbestos Exposure and Neoplasia. *JAMA*.

[14] Frost G., Harding A.-H., Darnton A., McElvenny D., Morgan D. (2008). Occupational exposure to asbestos and mortality among asbestos removal workers: a poisson regression analysis. *British Journal of Cancer*.

[15] Bianchi C., Bianchi T. (2007). Malignant mesothelioma: global incidence and relationship with asbestos. *Industrial Health*.

[16] McCormack V., Peto J., Byrnes G., Straif K., Boffetta P. (2012). Estimating the asbestos-related lung cancer burden from mesothelioma mortality. *British Journal of Cancer*.

[17] (2006). Elimination of Asbestos Related Disease. http://apps.who.int/iris/bitstream/10665/69479/1/WHO_SDE_OEH_06.03_eng.pdf.

[18] http://www.idescat.cat/emex/?id=081803#h1000000

[19] Tarrés J., Abós-Herràndiz R., Albertí C. (2009). Enfermedad por amianto en una población próxima a una fábrica de fibrocemento. *Archivos de Bronconeumología*.

[20] Rosell-Murphy M., Abás-Herrndiz R., Tarrés J. (2010). Prospective study of asbestos-related diseases incidence cases in primary health care in an area of Barcelona province. *BMC Public Health*.

[21] American Thoracic Society Documents (2004). Diagnosis and initial management of non-malignant diseases related to asbestos. *American Journal of Respiratory and Critical Care Medicine*.

[22] Abós-Herràndiz R., Puigdefàbregas-Serra A., Gispert-Magaroles R. Classification and codes of asbestos related diseases: from a small scale to a global view. http://www.who.int/classifications/network/PosterBooklet.zip?ua=1.

[23] Rothamn K. J., Greenland S., Rothman K. J., Greenland S. (1998). Causation and causal inference. *Modern Epidemiology*.

[24] Kaplan E. L., Meier P. (1958). Nonparametric estimation from incomplete observations. *Journal of the American Statistical Association*.

[25] Fine J. P., Gray R. J. (1999). A proportional hazards model for the subdistribution of a competing risk. *Journal of the American Statistical Association*.

[26] Lau B., Cole S. R., Gange S. J. (2009). Competing risk regression models for epidemiologic data. *American Journal of Epidemiology*.

[27] Greenland S., Rothman K. J., Greenland S. (1998). Application of stratified analysis methods. *Modern Epidemiology*.

[28] Ley orgánica 15/1999, de 13 de diciembre, de protección de datos de carácter personal. https://www.boe.es/boe/dias/1999/12/14/pdfs/A43088-43099.pdf.

[29] Pairon J.-C., Laurent F., Rinaldo M. (2013). Pleural plaques and the risk of pleural mesothelioma. *Journal of the National Cancer Institute*.

[30] Kumagai S., Kurumatani N. (2009). Asbestos fiber concentration in the area surrounding a former asbestos cement plant and excess mesothelioma deaths in residents. *American Journal of Industrial Medicine*.

[31] Dodoli D., Nevo M. D., Fiumalbi C. (1992). Environmental household exposures to asbestos and occurrence of pleural mesothelioma. *American Journal of Industrial Medicine*.

[32] Ferrante D., Bertolotti M., Todesco A., Mirabelli D., Terracini B., Magnani C. (2007). Cancer mortality and incidence of mesothelioma in a cohort of wives of asbestos workers in Casale Monferrato, Italy. *Environmental Health Perspectives*.

[33] Helland Å., Solberg S., Brustugun O. T. (2012). Incidence and survival of malignant pleural mesothelioma in Norway: a population-based study of 1686 cases. *Journal of Thoracic Oncology*.

[34] Ameille J., Brochard P., Letourneux M., Paris C., Pairon J.-C. (2011). Asbestos-related cancer risk in patients with asbestosis or pleural plaques. *Revue des Maladies Respiratoires*.

[35] Lee L. J.-H., Chang Y.-Y., Liou S.-H., Wang J.-D. (2012). Estimation of benefit of prevention of occupational cancer for comparative risk assessment: Methods and examples. *Occupational and Environmental Medicine*.

[36] Elkiran E. T., Kaplan M. A., Sevinc A. (2012). Multicentric study on malignant pleural mesothelioma in Turkey: Clinicopathologic and survival characteristics of 282 patients. *Medical Oncology*.

[37] Rothman K. J., Greenland S., Rothman K. J., Greenland S. (1998). Cohort studies. *Modern Epidemiology*.

[38] Schreier H. (1989). *Asbestos in The Natural Environment*.

[39] Mata-Perelló J. M. (1990). Els minerals de Catalunya. Jaciments d’amiant a Catalunya. Barcelona. *Institut d'Estudis Catalans*.

[40] López-Abente G., Ramis R., Pollán M. (2007). Área de Epidemiología Ambiental y Cáncer del Centro Nacional de Epidemiología. *Atlas municipal de mortalidad por cáncer 1989-1998*.

[41] Hosoda Y., Hiraga Y., Sasagawa S. (2008). Railways and asbestos in Japan (1928-1987) - epidemiology of pleural plaques, malignancies and pneumoconioses. *Journal of Occupational Health*.

[42] Pass H. I. (2002). Pleural mesothelioma in 2002: Going somewhere very slowly. *Journal of Thoracic and Cardiovascular Surgery*.

[43] Campbell N. P., Kindler H. L. (2011). Update on malignant pleural mesothelioma. *Seminars in Respiratory and Critical Care Medicine*.

[44] Don de Savigny, Taghreed A. (2009). *Systems thinking for health systems strengthening. Alliance for Health Policy and systems Research*.

[45] Ross M., Langer A. M., Nord G. L. (2008). The mineral nature of asbestos. *Regulatory Toxicology and Pharmacology*.

[46] Ramazzini C. (1999). Editorial Board of the American Journal of Industrial Medicine. *American Journal of Industrial Medicine*.

[47] Park E.-K., Takahashi K., Jiang Y., Movahed M., Kameda T. (2012). Elimination of asbestos use and asbestos-related diseases: an unfinished story. *Cancer Science*.

